# RNA polymerase II subunit D is essential for zebrafish development

**DOI:** 10.1038/s41598-020-70110-1

**Published:** 2020-08-06

**Authors:** Masanari Maeta, Miku Kataoka, Yusuke Nishiya, Kazutoyo Ogino, Makoto Kashima, Hiromi Hirata

**Affiliations:** Department of Chemistry and Biological Science, College of Science and Engineering, Aoyama Gakuin University, Sagamihara, 252-5258 Japan

**Keywords:** Embryogenesis, Developmental biology

## Abstract

DNA-directed RNA polymerase II (pol II) is composed of ten core and two dissociable subunits. The dissociable subcomplex is a heterodimer of Rpb4/Polr2d and Rpb7/Polr2g, which are encoded by *RPB4/polr2d* and *RPB7/polr2g* genes, respectively. Functional studies of Rpb4/Polr2d in yeast have revealed that Rpb4 plays a role primarily in pol II-mediated RNA synthesis and partly in various mRNA regulations including pre-mRNA splicing, nuclear export of mRNAs and decay of mRNAs. Although Rpb4 is evolutionally highly conserved from yeast to human, it is dispensable for survival in budding yeast *S. cerevisiae*, whereas it was indispensable for survival in fission yeast *S. pombe*, slime molds and fruit fly. To elucidate whether Rpb4/Polr2d is necessary for development and survival of vertebrate animals, we generated *polr2d*-deficient zebrafish. The *polr2d* mutant embryos exhibited progressive delay of somitogenesis at the onset of 11 h postfertilization (hpf). Mutant embryos then showed increased cell death at 15 hpf, displayed hypoplasia such as small eye and cardiac edema by 48 hpf and prematurely died by 60 hpf. In accordance with these developmental defects, our RT-qPCR revealed that expression of housekeeping and zygotic genes was diminished in mutants. Collectively, we conclude that Rpb4/Polr2d is indispensable for vertebrate development.

## Introduction

DNA-directed RNA polymerase II, which is often referred to as Pol II, is the RNA synthesis enzyme that is composed of twelve different subunits designated as Rpb1 to Rpb12. Rpb1 is the largest subunit with respect to molecular weight, while Rpb12 is the smallest. Among them, ten subunits form the catalytic core and the remaining two subunits Rpb4 and Rpb7 form dissociable external subcomplex^1,2^. The Rpb4/Rpb7 heterodimer plays an important role in promoter-dependent initiation of transcription of protein-coding genes as a cofactor of Pol II^3^. In addition to this function, Rpb4/Rpb7 contributes to various RNA metabolism functions including transcription-coupled DNA repair and pre-mRNA splicing in the nucleus as well as nuclear export of mRNAs, initiation of translation and decay of mRNAs in the cytoplasm^4–7^. In agreement with these functions, Rpb4/Rpb7 heterodimer shuttles between the nucleus and the cytoplasm^8^.

The physiological role of Rpb4/Rpb7 has been investigated in several organisms especially in yeast. In *Saccharomyces cerevisiae* (*S. cerevisiae*), Rpb4 was required for growth at high and low temperatures, but a deletion of the *RPB4* gene was not lethal at regular temperature^9^. A transcriptome analysis revealed that Rpb4 was involved in transcription of many genes in *S. cerevisiae*^10^. On the other hand, Rpb4 was indispensable for cell survival in *Schizosaccharomyces pombe* (*S. pombe*) at any temperature^11^. Similarly, *RPB4* gene was essential for survival in slime molds (*Dictyostelium dicoideum*)^12^. The Rpb4 protein in fruit fly (*Drosophila melanogaster*) was expressed with a transcriptional adaptor protein Ada2a by a bi-cistronic operon. Null fly mutants for both *Rpb4* and *Ada2a* genes died during an early larval L1 stage^13^. In human and other vertebrates, Rpb4 protein was referred to as RNA polymerase II subunit D (Polr2d) and the *RPB4* gene was renamed to *POLR2D*. The shRNA-mediated knockdown of *POLR2D* in several human cell lines suggested that *POLR2D* is necessary for survival in cultured human cells^14^. However, in vivo function and necessity of *POLR2D* remains unsolved in vertebrates, because *POLR2D* knockout vertebrate animals have not been generated.

Zebrafish (*Danio rerio*) have several advantages for the analysis of embryogenesis in vertebrates^15^. All stages of development occur externally and rapidly, with most organs formed within 72 h of fertilization^16^. The fast pace of development allows us to analyze embryogenesis in live zebrafish. The embryos are transparent, which makes them amenable for whole embryo imaging of cell morphology, cell migration and cell death. Recent development of CRISPR/Cas9 technology enabled quick generation of knockout zebrafish for loss of function studies. Collectively, zebrafish is an emerging vertebrate model to study development as well as pathology of congenital disorders.

To analyze physiologically-relevant function of Polr2d in vertebrate development, we employed CRISPR/Cas9 method in zebrafish and generated *polr2d*-deficient zebrafish. The *polr2d* mutants initially showed retardation of somitogenesis, eventually exhibited increased cell death and finally displayed severe hypoplasia, resulting in premature death. Our RNA analysis revealed that mRNA levels were significantly affected in *polr2d* mutant embryos. Taken together, we demonstrated that Rpb4/Polr2d contributes to mRNA regulation of housekeeping and zygotic genes and is essential for vertebrate development.

## Results

### Generation of *polr2d*-deficient zebrafish

**Figure 1:**
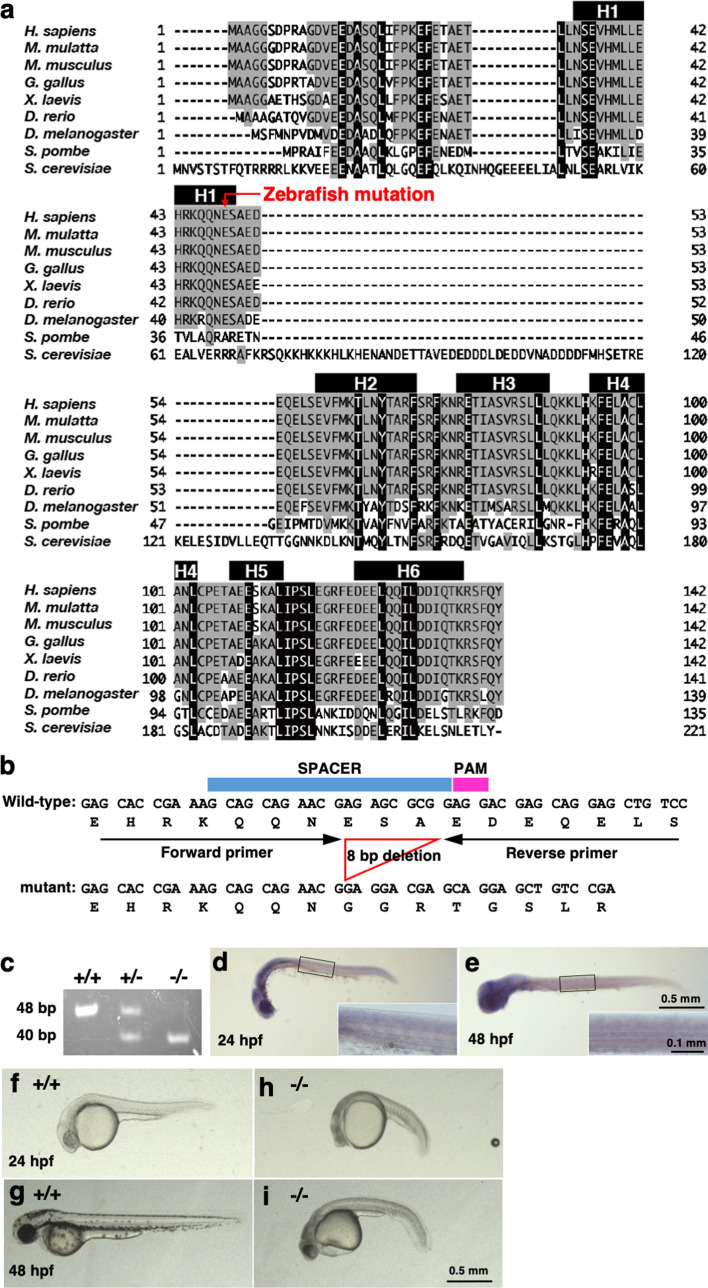
Generation of zebrafish *polr2d* mutant zebrafish. (**a**) Multiple amino acid sequence alignment of Polr2d/Rpb4 protein. Black and grey colors respectively indicate completely and highly conserved residues among eukaryotes. Black boxes H1–H6 mark the region of helix domain 1–6. The position of frame-shift mutation in zebrafish is indicated by a red arrow in H1. Following NCBI data were used: human (*H. sapines*) NP_004796; rhesus monkey (*Macaca mulatta*) NP_001254716; mouse (*Mus musculus*) NP_081278; chicken (*Gallus gallus*) NP_001264338; african clawed frog (*Xenopus laevis*) NP_001087315; zebrafish (*Danio rerio*) NP_001002317; fruit fly (*Drosophila melanogaster*) NP_001014633; fission yeast (*Schizosaccharomyces pombe*) NP_595415; budding yeast (*Saccharomyces cerevisiae*) NP_012395. (**b**) A genomic deletion and a consequent frame-shift mutation in zebrafish *polr2d*. The 8-bp deletion was generated in the spacer sequence of the CRISPR. The position of forward and reverse primers for genotyping are indicated. (**c**) Genotyping PCR. The size of wild-type and mutant bands were 48 and 40 bp, respectively. (**d**, **e**) Spatial and temporal expression of *polr2d*. Whole-mount in situ hybridization of *polr2d* in zebrafish embryos at 24 hpf (**d**) and 48 hpf (**e**). Magnified views of the boxed region indicate *polr2d* expression in the spinal cord. (**f**–**i**) Images of *polr2d* mutants. The *polr2d* mutants showed developmental defects at 24 hpf (**h**) and 48 hpf (**i**) compared to wild-type (**f**, **g**). Note that small eye and cardiac edema were evident in mutants at 48 hpf.

To investigate *polr2d* gene in zebrafish, we cloned full length *polr2d* cDNA and identified 141 amino acid sequence. An amino acid alignment of Polr2d/Rpb4 protein from many organisms showed that Polr2d is conserved from yeast to human, especially among vertebrate animals (Fig. 1a). We designed a spacer and a PAM for CRISPR/Cas9-mediated genome editing in the conserved helix-1 domain of *polr2d* gene, injected guide RNA along with Cas9 mRNA into fertilized zebrafish embryos and then obtained an 8-bp deletion allele of *polr2d* (Fig. 1b). This deletion caused a frameshift in the helix-1 domain, thereafter adding 30 amino acids followed by a stop codon. Genomic PCR enabled reliable genotyping of wild-type (+ / +), heterozygous mutant (+/−) and homozygous mutant (−/−) embryos (Fig. 1c).

To assess spatial and temporal expression of *polr2d*, we performed whole mount in situ hybridization using full length *polr2d* cRNA as a probe. At 24 and 48 hpf, *polr2d* was ubiquitously expressed with intense signals in the brain and the spinal cord (Fig. 1d,e) in accordance with a publicly available expression data^17,18^.

Heterozygous mutant embryos showed no developmental defects and grew up to become normal adults. By crossing heterozygous mutant carrier fish, we obtained homozygous mutant embryos at a ratio of one quarter (27.1%, 26/96). Hereafter, we simply refer to homozygous mutants as mutants. The mutants showed apparent delay of embryogenesis at 24 hpf and then exhibited developmental hypoplasia such as small eye and cardiac edema at 48 hpf (Fig. 1f–i). All of the mutant embryos died between 48 and 60 hpf. These hypoplasia at 48 hpf were cancelled by injecting wild-type *polr2d* mRNA into mutants at 1–2 cell stage (81% rescue, 17/21), thereby confirming that developmental defects in mutants were attributable to the loss of Polr2d function. These results indicate that *polr2d* is essential for zebrafish embryogenesis.

### Delay of development in *polr2d* mutant zebrafish

**Figure 2:**
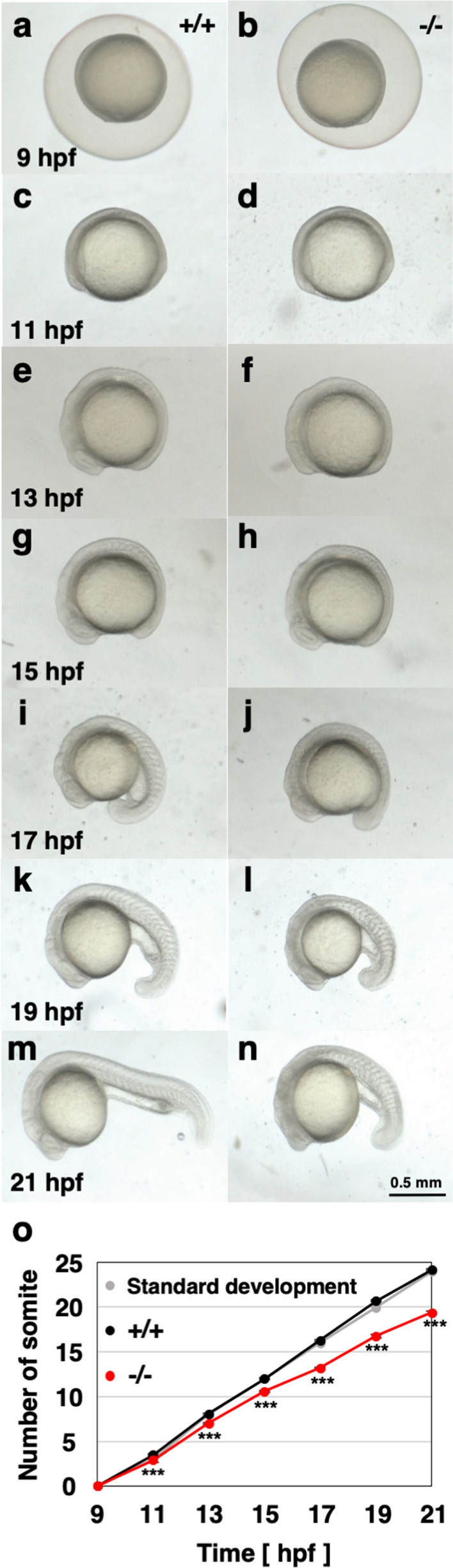
Delay of development in *polr2d* mutants. (**a**–**n**) Images of wild-type and mutant zebrafish embryos. (**O**) The increase of somite number during embryogenesis. In standard developmental condition at 28.5 °C, wild-type embryos form 3 somites per hour at 10–12 hpf and 2 somites per hour at 12–24 hpf (grey). Wild-type embryos showed somite formation at normal pace (n = 39; black). Mutants showed a delay of somitogenesis at the onset of 11 hpf (n = 33, red).

To detail the delay of embryogenesis in *polr2d* mutants, we assessed precise developmental stages of mutant embryos by counting somite numbers. In standard zebrafish development at 28.5 °C, somite formation is initiated at the onset of 10 hpf with the rate of 3 somites per hour. After 12 hpf (6 somite), the rate becomes 2 somites per hour and somitogenesis continues up to 24 hpf (30 somites). We obtained embryos by crossing heterozygous mutant carrier fish, counted the number of somite in each embryo every 2 h from 9 to 21 hpf and checked genotype for each. At 9 hpf when somitogenesis is not yet initiated, wild-type and mutant embryos were indistinguishable in appearance (Fig. 2a,b). Although wild-type and mutant embryos looked similar at 11 hpf, the number of somite in mutants was significantly lower than that in wild-type (wild-type: 3.5 ± 0.1; mutant: 2.9 ± 0.2; *P* < 0.001; Fig. 2c,d,o). This difference increased along with somitogenesis (13 hpf wild-type: 8.1 ± 0.1; 13 hpf mutant: 7.0 ± 0.1; 15 hpf wild-type: 12.0 ± 0.1; 15 hpf mutant: 10.6 ± 0.1; 17 hpf wild-type: 16.3 ± 0.1; 17 hpf mutant: 13.2 ± 0.1; 19 hpf wild-type: 20.7 ± 0.1; 19 hpf mutant: 16.8 ± 0.2; 21 hpf wild-type: 24.2 ± 0.1; 21 hpf mutant: 19.4 ± 0.2; all stages *P* < 0.001; Fig. 2e–n). The pace of developmental in heterozygous mutant embryos was indistinguishable from that in wild-type embryos (data not shown). These results indicate that progressive retardation of embryogenesis appears at the onset of 11 hpf in *polr2d* mutants. We also noticed that head region in mutants became less transparent after 19 hpf. This typically suggests that many neuronal cells undergo cell death in *polr2d* mutants.

### Increased cell death in *polr2d* mutants

**Figure 3:**
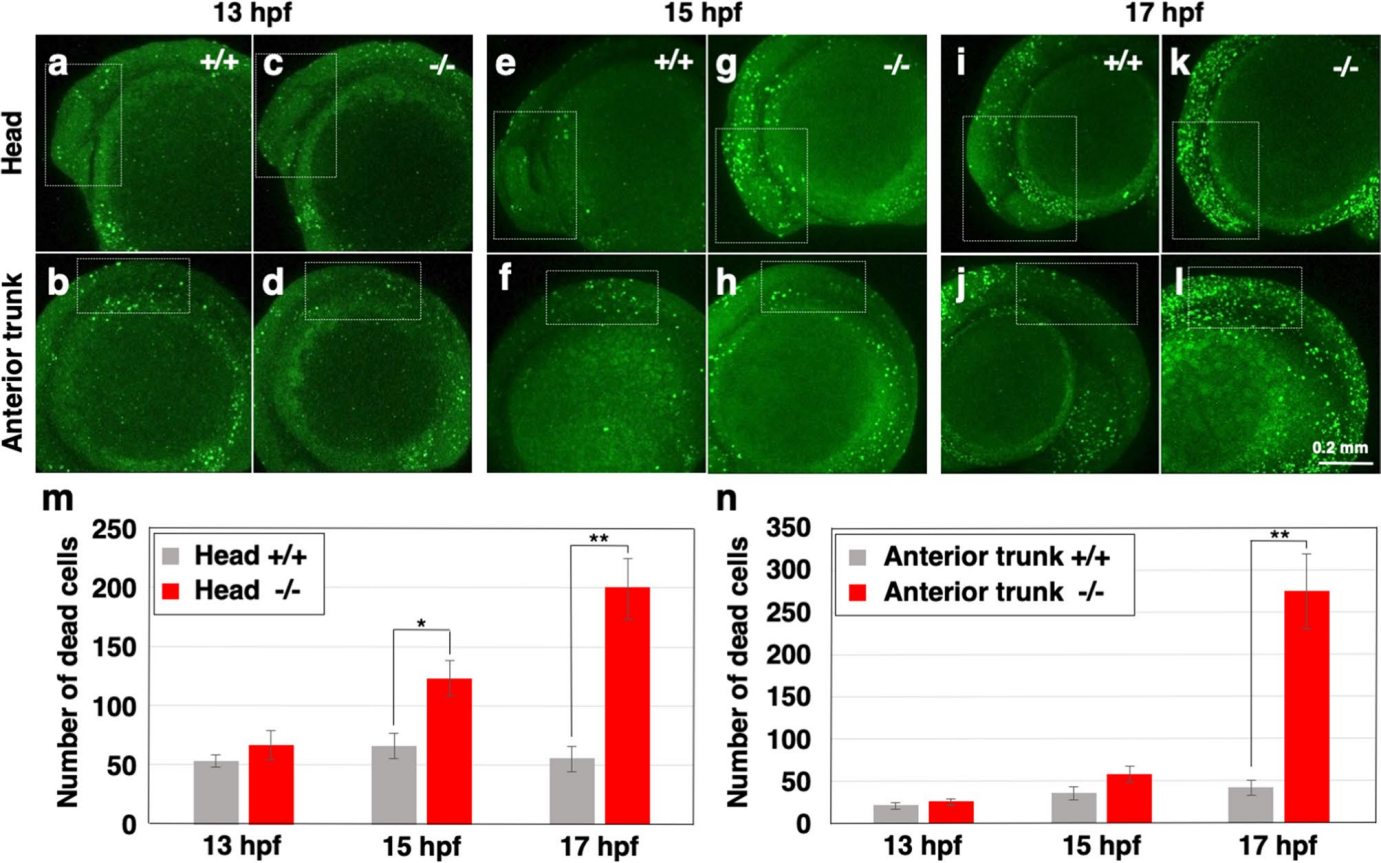
Increase of cell death in *polr2d* mutants. (**a**–**l**) Images of acridine orange-labeled dead cells in zebrafish embryos. White dotted boxes mark the head (anterior to midbrain/hindbrain boundary; **a**, **b**, **e**, **f**, **i**, **j**) and anterior trunk (somite 1–4; **c**, **d**, **g**, **h**, **k**, **l**) regions. (**m**, **n**) The number of dead cells in the head (**m**) and anterior trunk (**n**) regions. Note that cell death in mutants increased at the onset of 15 hpf in the head and at the onset of 17 hpf in the anterior trunk. n = 7 for each sample set.

To address whether cell death is accelerated in mutants, we counted the number of cells that undergo cell death during embryogenesis. By immersing live zebrafish embryos in acridine orange-containing solution, we could fluorescently label and count dead cells at each time point^19^. Samples were then subjected to genotyping. At 13 hpf, the numbers of dead cells in the head (anterior to midbrain/hindbrain boundary) and the anterior trunk (somite 1–4) regions were comparable between wild-type (head 52.8 ± 5.0; anterior trunk 21.2 ± 3.7; Fig. 3a,b) and mutants (head 65.6 ± 11.9, *P* = 0.30; anterior trunk 25.0 ± 4.1, *P* = 0.51; Fig. 3c,d,m,n). At 15 hpf, fluorescence-positive dead cells in mutant head (121.3 ± 15.0; Fig. 3g) were significantly larger in number than those in wild-type head (65.9 ± 10.5, *P* < 0.05; Fig. 3e), whereas the dead cells in the anterior trunk region were similarly seen in wild-type (35.1 ± 7.6; Fig. 3f) and mutants (53.5 ± 9.3, *P* = 0.08; Fig. 3h). At 17 hpf, the number of dead cells in mutants (head 198.4 ± 25.2; anterior trunk 274.8 ± 44.4; Fig. 3k,l) was larger than that in wild-type (head 54.3 ± 10.7, *P* < 0.01; anterior trunk 41.9 ± 8.8, *P* < 0.01; Fig. 3i,j) at both head and anterior trunk regions. The cell death in heterozygous mutant embryos was similarly seen as in wild-type embryos (data not shown). These results indicate that cell death is accelerated in *polr2d* mutants during embryogenesis.

### Gene expression is affected in *polr2d* mutants

**Figure 4:**
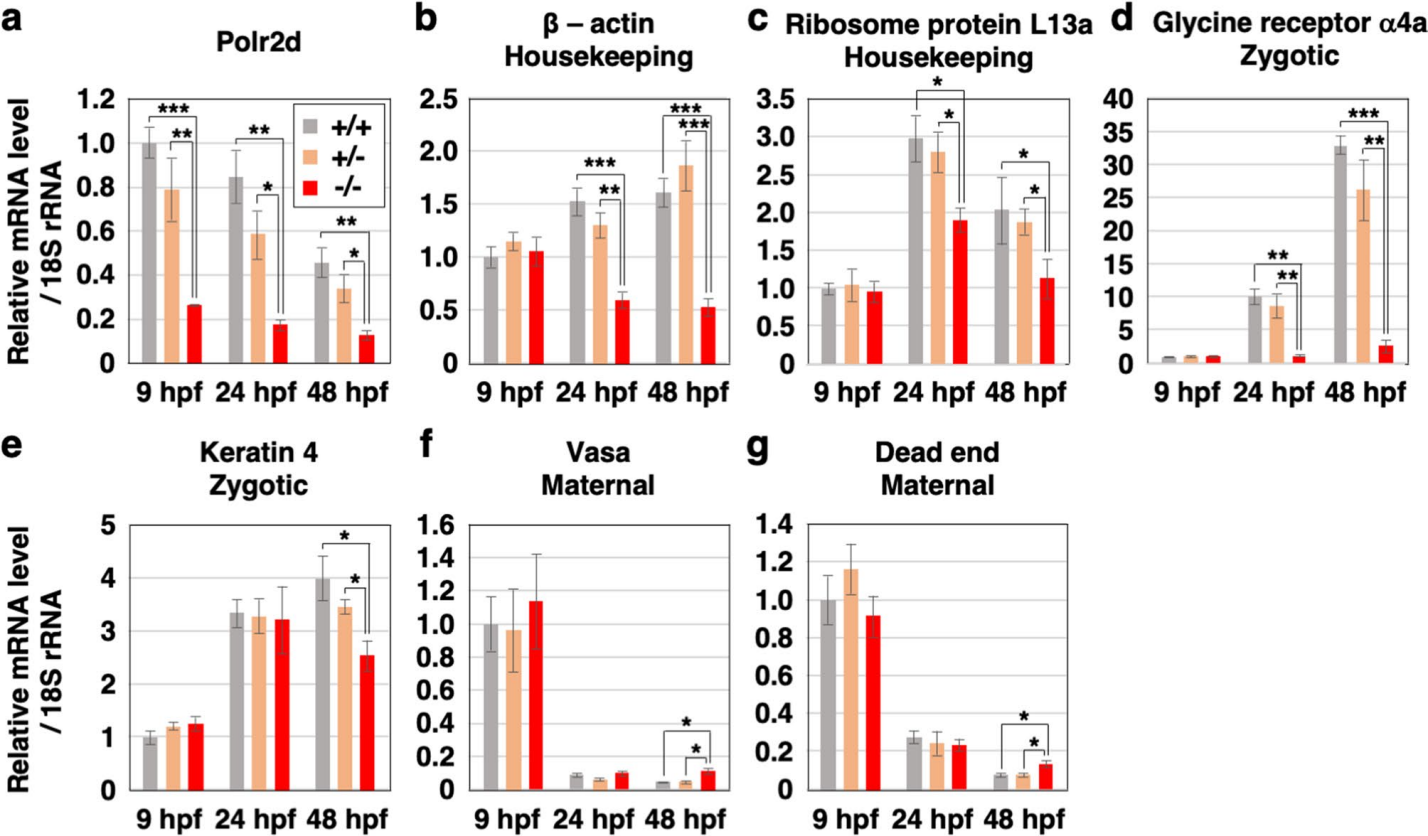
The mRNA levels are affected in *polr2d* mutants. Expression of several genes relative to 18S rRNA level in wild-type (+/+), heterozygous mutant (+/−) and homozygous mutant (−/−) embryos. The Polr2d gene (*polr2d*: **a**). Housekeeping genes: β-actin (*actb1*: **b**) and ribosome protein L13a (*rpl13a*: **c**). Zygotic genes: glycine receptor α4a subunit (*glra4a*: **d**) and keratin 4 (*krt4*: **e**). Maternal genes: DEAD-box RNA helicase vasa (*vasa*: **f**) and RNA-binding protein dead end (*dnd*: **g**). Note that mRNA levels of housekeeping and zygotic genes but not maternal genes were reduced in mutants at 24 and 48 hpf. n = 5 for each sample set.

Since Polr2d/Rpb4 plays important roles in Pol II-dependent synthesis of mRNA in yeast, we assessed mRNA levels of several genes in wild-type and *polr2d* mutants. We collected total RNAs and genomic DNAs from individual embryos of heterozygous carrier crosses and performed RT-qPCR and genotyping. We then compared mRNA levels relative to 18S rRNA levels, since 18S rRNA is transcribed by pol I, which does not require Polr2d/Rpb4 for rRNA synthesis, and is suitable for the internal standard in this case^20^. The mRNA levels of *polr2d* in mutants were diminished at 9, 24 and 48 hpf compared to those in wild-type and heterozygous mutant embryos (Fig. 4a). Expression of housekeeping genes such as β-actin (*actb1*) and ribosomal protein L13a (*rpl13a*) in mutants were initially comparable to those in wild-type siblings at 9 hpf (Fig. 4b,c). But they eventually decreased compared to those in wild-type and heterozygous mutant embryos at 24 and 48 hpf. Similarly, expression of zygotic genes such as glycine receptor α4a subunit (*glra4a*) and keratin 4 (*krt4*) in mutants was unaffected by 9 hpf but then reduced in comparison to wild-type and heterozygous mutant embryos by 48 hpf (Fig. 4d, e). The levels of maternal transcripts such as DEAD-box RNA helicase vasa (*vasa*) and RNA-binding protein dead end (*dnd*) were comparable between wild-type siblings and mutant embryos at 9 and 24 hpf (Fig. 4f,g). In both cases, mRNA levels in mutants did not become lower and rather became higher than those in wild-type and heterozygous mutant embryos at 48 hpf. These results confirm that Polr2d/Rpb4 is involved in Pol II-mediated transcription of housekeeping and zygotic genes during embryogenesis.

## Discussion

In this study, we generated *polr2d* knockout vertebrate animals for the first time and demonstrated that *polr2d* mutant zebrafish showed initially the delay of somite formation, eventually the increase of cell death and finally developmental hypoplasia and premature death. Expression of housekeeping and zygotic genes was severely affected in mutants. Taken together, we conclude that Polr2d/Rpb4, which is encoded by *polr2d/RPB4* gene, is essential for mRNA regulation and embryogenesis in vertebrates.

### Loss of *polr2d* and lethality

To investigate the physiological necessity of Polr2d/Rpb4, disruption of *polr2d/RPB4* gene has been assayed in several organisms ranging from yeast to invertebrate animals. All of the *polr2d/RPB4*-deficient organisms were lethal, except for *RPB4* mutant *S. cerevisiae* that could grow at normal temperature^9^. In vertebrates, our *polr2d* mutant zebrafish were embryonic lethal. A previous study of retrovirus-mediated mutagenesis showed that zebrafish embryos homozygously carrying a retroviral insertion in an intron of *polr2d* gene (hi2718b) exhibited the increase of neuronal cell death^21,22^. Since this apoptotic phenotype in hi2718b embryos is similar to that in our *polr2d* mutants, hi2718b defects are attributable to the *polr2d* deficiency, supporting our notion that *polr2d* is indispensable for zebrafish development. In cultured human cells, cell proliferation was severely impaired by shRNA-mediated *POLR2D/RPB4* knockdown^14^. Collectively, we can conclude that *POLR2D/RPB4* gene is essential for survival in eukaryotes with an exception of the *S. cerevisiae* case. Interestingly, only *S. cerevisiae* have 11-amino acids insertion and 60-amino acids insertion before helix-1 domain and in between helix-1 and helix-2 domains, respectively^9^. Although the significance of these *S. cerevisiae*-specific insertion is unclear, this organism might have divided Rpb4 function and distributed it to Rpb4 and the other proteins.

### mRNA defects in zebrafish *polr2d* mutants

In the earliest stages of animal development, a fertilized egg initiates cell cleavages. Transcription of zygotic genes is quiescent immediately after fertilization and then is activated in a phase called maternal-to-zygotic transition^23,24^. In zebrafish, this transition occurs at 2–4 hpf^25,26^. Although we anticipated the impairment of transcription during maternal-to-zygotic transition in mutants, our RT-qPCR analyses revealed that mRNA levels in mutants were not globally affected at 9 hpf. We assume that *polr2d* is a housekeeping gene that is maternally transcribed in oocytes. Therefore, *polr2d* mutants likely have maternal reserves of normal *polr2d* mRNAs that enabled the synthesis of normal Polr2d protein during early embryogenesis. As a consequence, mRNA levels in mutant embryos were mostly unaffected by 9 hpf. But after this stage, mutants could no longer sustain Pol II-dependent transcription due to the running out of Polr2d protein, leading to progressive defects of embryogenesis.

Despite that mRNA levels of housekeeping and zygotic genes were maintained in mutants at 9 hpf, *polr2d* mRNA was significantly reduced at the same time. This specific elimination of *polr2d* mRNA was probably caused not by the impairment of Pol II-dependent transcription but rather by nonsense-mediated mRNA decay. The nonsense-mediated mRNA decay is an active degradation system of abnormal mRNAs that harbor premature nonsense codons^27^. Since *polr2d* gene has four exons and the frame-shift mutation generating a downstream nonsense codon was located in the second exon, mutant *polr2d* mRNAs can be the target of nonsense-mediated decay. This accounts for the early specific loss of *polr2d* mRNA in mutants.

### Progressive developmental defects in *polr2d* mutants

We could observe a significant delay of development in *polr2d* mutants at the onset of 11 hpf. The degree of the delay was only 0.6 somite that corresponds to 12 min at 11 hpf. The degree of the delay became larger during somite formation, which took place in an anterior to posterior wave (13 hpf: 1.1 somite = 33 min; 15 hpf: 1.4 somite = 42 min; 17 hpf: 3.1 somite = 93 min; 19 h: 3.9 somite = 117 min; 21 hpf: 4.8 somite = 144 min). This progressive delay of developmental was followed by the increase of cell death at the onset of 15 hpf. The cell death accelerated progressively in an anterior to posterior direction, because cell death is triggered by the failure of neuronal differentiation, which proceeds in the same direction along with body extension^28,29^. Then, mutant embryos displayed reduced transparency and developmental hypoplasia such as small eye and cardiac edema at 24 and 48 hpf. Small eye and cardiac edema are typical phenotypes of gross developmental defects in zebrafish^30,31^. Collectively, *polr2d* mutation caused sequential developmental defects: the delay of development, the increase of cell death and developmental hypoplasia.

## Materials and methods

### Animals

Zebrafish (*Danio rerio*) AB line was purchased from Zebrafish International Resource Center (https://zebrafish.org/home/guide.php). They were reared and maintained at 28.5 °C under a 14 h light and 10 h dark photoperiod and fed twice a day as in the regular care and maintenance protocol^32^. The *polr2d* mutant allele that harbors 8-bp deletion was generated in this study and designated as *agu8* by the Zebrafish Nomenclature Committee in ZFIN (https://zfin.org). This allele is available from National BioResource Project Zebrafish (https://shigen.nig.ac.jp/zebra/). All of the *polr2d* homozygous mutants used in this study was obtained by crossing heterozygous mutant carrier fish that was back-crossed to wild-type zebrafish AB line at least twice.

### Cloning of full length zebrafish *polr2d* cDNA

Total RNA was extracted from embryos of zebrafish AB line using Sepasol RNA II Super (Nacalai Tesque). The Oligo(dT)18 Primer (Thermo Fisher Scientific), SuperScript IV Reverse Transcriptase (Thermo Fisher Scientific), *polr2d* cloning primers and Phusion DNA polymerase (Thermo Fisher Scientific) were used for RT-PCR as described previously^33^. The *polr2d* cDNA was cloned into pCR4Blunt-TOPO vector (Thermo Fisher Scientific) and pCS2 + expression vector. The *polr2d* cloning primer sequences are listed in Table 1.

**Table 1:** Oligonucleotide primes used in this study.

Oligonucleotide primer	Sequence
***polr2d ***cloning	
Forward	GAGAGAGAGAATTCGCCGCCACCATGGCGGCGGCAGGAGCAAC
Reverse	GTGTGTGTCTCGAGTCAGTACTGGAAGCTGCGTTTGGTCTG
***polr2d ***CRISPR	
Forward	TAGGGCAGCAGAACGAGAGCGCGG
Reverse	AAACCCGCGCTCTCGTTCTGCTGC
***polr2d ***genotyping	
Forward	GCACCGAAAGCAGCAGAACG
Reverse	GACAGCTCCTGCTCGTCCTC
***polr2d ***RT-qPCR	
Forward	CCAGCTCATGTTTCCTAAAGAGTTTG
Reverse	GCAGCAGCAAACTGCGC
***actb1 ***RT-qPCR	
Forward	ACTTTGAGCTCCTCCACACG
Reverse	AGTGCGGCAATTTCATCATC
***rpl13a ***RT-qPCR	
Forward	CTGGAGGACTGTAAGAGGTATGC
Reverse	ACGCACAATCTTGAGAGCAG
***glra4a ***RT-qPCR	
Forward	TGCAGGTTGCGGATGACTTG
Reverse	AGGCTGGGGATGTACATCTG
***krt4 ***RT-qPCR	
Forward	AGACCCTCAACAACCGCTTC
Reverse	GCATCGATGTTGGAACGTGT
***vasa ***RT-qPCR	
Forward	CCAGAGTCTGACACTCCATTAGCT
Reverse	GAGCACTGAAGGCAACTTCCTC
***dnd ***RT-qPCR	
Forward	ACCCAAGTCAATGGGCAGAG
Reverse	TACACGTCGTTCGGGATCTG
**18S rRNA RT-qPCR**	
Forward	CCCTTCCGTCAATTCCTTTAAG
Reverse	ATCAGATACCGTCGTAGTTCCG

### mRNA rescue

Capped, full-length zebrafish *polr2d* mRNA was synthesized from pCS2 + zPolr2d plasmids using the mMessage mMachine SP6 Transcription Kit (Thermo Fisher Scientific) as described previously^34^. The mRNA was microinjected into 1–2 cell stage embryos and the embryos were subjected for observation at 48 hpf as described previously^35^.

### Generation of *polr2d* mutants by CRISPR/Cas9

CRISPR/Cas9 targets were selected in exons 2 of zebrafish *polr2d* gene using a web-based free software CRISPRscan (https://www.crisprscan.org)^36^. To generate a gRNA construct, *polr2d* CRISPR forward and reverse oligonucleotides (Table 1) were annealed and subcloned into pDR274^37^. The gRNAs were synthesized from Hind III-digested pDR274-zPolr2d plasmids using MEGAshortscript T7 Transcription Kit (Thermo Fisher Scientific). The Cas9 RNA was synthesized from pCS2 + hSpCas9^38^, which contains the human codon–optimized *S. pyogenes* Cas9 cDNA^39^, using mMESSAGE mMACHINE SP6 Transcription Kit (Thermo Fisher Scientific). A solution containing 20 ng/μl gRNA and 200 ng/μl Cas9 RNA was injected into zebrafish embryos at the one-cell stage. To detect insertion/deletion mutations, the target regions were amplified by PCR and subjected to heteroduplex mobility analysis^40^. The 8-bp deletion was confirmed by sequencing after subcloning of the target region.

### Genotyping

The 8-bp deletion region of *polr2d* gene was amplified by genomic PCR using KAPA Taq Extra PCR Kit (Kapa Biosystems) in ProFlex PCR System (Thermo Fisher Scientific). Following program was used for amplification: 94 °C 2 min; 98 °C 10 s, 58 °C 20 s, 72 °C 30 s, 35 cycles; 72 °C 1 min; 4 °C forever. PCR products were separated by 15% polyacrylamide gel electrophoresis at 300 V for 90 min and the gel images were captured using the Printgraph AE-6933FXCF (Atto).

### Reverse transcription quantitative PCR (RT-qPCR)

The RT-qPCR was done as described previously^41^. Total RNA was extracted from zebrafish embryos using Sepasol RNA II Super (Nacalai Tesque). For reverse transcription, SuperScript IV Reverse Transcriptase (Thermo Fisher Scientific) and Random Primer Mix (New England Labs), the latter contains both random 6-mer and oligo-dT oligonucleotides, were used to synthesize cDNAs. The qPCR was carried out using gene-specific primers with a KAPA SYBR Fast qPCR Kit (Kapa Biosystems) on QuantStudio 5 Real-Time PCR System (Thermo Fisher Scientific). The 18S rRNA was used as an internal control. Each qPCR amplification was performed in triplicate and the data were analyzed using the 2^−ΔΔCt^ program as described previously^42^. The gene-specific primer sequences are listed in Table 1.

### In situ hybridization

In situ hybridization of wholemount zebrafish embryos with a digoxigenin-labeled antisense RNA probe was performed as described previously^43^. A *polr2d* probe covering the full coding sequence was used as a probe.

### Counting the number of somite

Zebrafish embryos obtained by crossing heterozygous mutant carrier fish were incubated in embryo medium in individual wells of 48-well plate (As One) at 28.5 °C. At each time point after fertilization, images of whole embryos were captured under a stereo microscope SZX16 (Olympus) as described previously^44^. The number of somite in each embryo was visually counted. Each embryo was subjected to genotyping later.

### Acridine orange labeling of dead cells

Live zebrafish embryos were immersed in embryo medium containing 5 μg/ml acridine orange (Sigma-Aldrich) at 28.5 °C for 60 min and washed in embryo medium for three times. Fluorescent images of labeled embryos were captured using a confocal microscopy TCS SP5 II (Leica) at each time point. The number of fluorescence-positive cells were counted.

### Statistics

Quantitative data were given as mean ± SEM. All error bars in graphs represent the SEM values. Statistical significance was determined using the two-tailed Student’s t test. Asterisks show *P* value ranges; *P < 0.05; **P < 0.01; ***P < 0.001. The sample numbers are indicated in Figure legends.

### Ethics statement

All animal experiments were approved by Animal Care and Use Committee of Aoyama Gakuin University (A9/2020) and performed in accordance with guidelines approved by the committee.

## Supplementary information

Supplementary Information.
