# Supplementary material for: RNA polymerase II subunit D is essential for zebrafish development
Source: Sci Rep. 2020 Aug 6;10:13213. doi: 10.1038/s41598-020-70110-1 (PMC7413394; doi:10.1038/s41598-020-70110-1)

RNA polymerase II subunit D is essential for zebrafish development

Masanari Maeta, Miku Kataoka, Yusuke Nishiya, Kazutoyo Ogino, Makoto Kashima and Hiromi Hirata*

Department of Chemistry and Biological Science, College of Science and Engineering, Aoyama Gakuin University, Sagamihara 252-5258, Japan

***Correspondence:** Hiromi Hirata hihirata@chem.aoyama.ac.jp

Supplementary Information

The original uncropped gel image of Figure 1c.

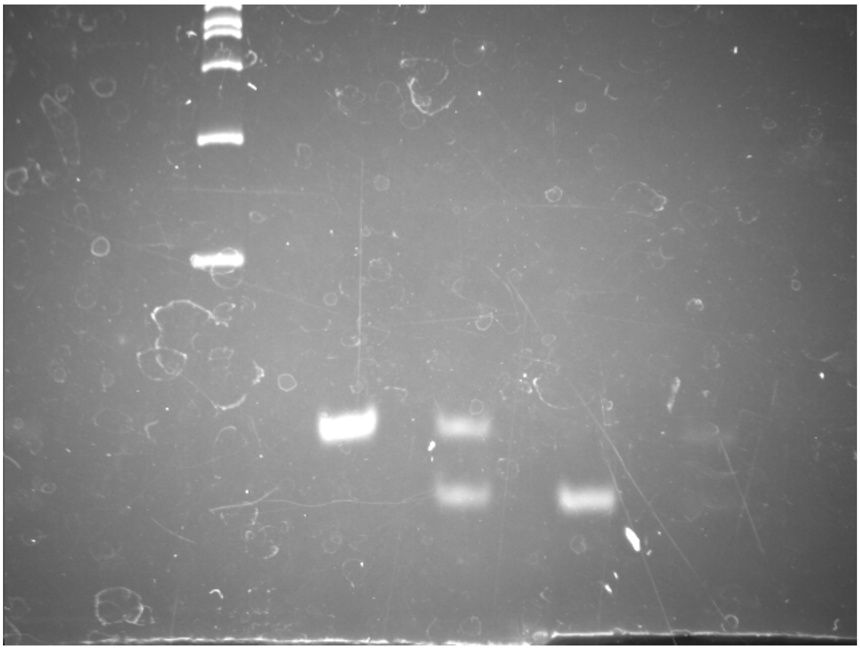

Supplement: Supplementary file 1 — Supplementary Information. [file 41598_2020_70110_MOESM1_ESM.pdf]
